# Controllable multichannel acousto-optic modulator and frequency synthesizer enabled by nonlinear MEMS resonator

**DOI:** 10.1038/s41598-021-90248-w

**Published:** 2021-05-25

**Authors:** Gayathri Pillai, Sheng-Shian Li

**Affiliations:** 1 Institute of NanoEngineering and MicroSystems, National Tsing Hua University, Hsinchu City, Taiwan; 2 Department of Power Mechanical Engineering, National Tsing Hua University, Hsinchu City, Taiwan

**Keywords:** Energy science and technology, Engineering, Optics and photonics, Physics

## Abstract

Nonlinear physics-based harmonic generators and modulators are critical signal processing technologies for optical and electrical communication. However, most optical modulators lack multi-channel functionality while frequency synthesizers have deficient control of output tones, and they additionally require vacuum, complicated setup, and high-power configurations. Here, we report a piezoelectrically actuated nonlinear Microelectromechanical System (MEMS) based Single-Input-Multiple-Output multi-domain signal processing unit that can simultaneously generate programmable parallel information channels (> 100) in both frequency and spatial domain. This significant number is achieved through the combined electromechanical and material nonlinearity of the Lead Zirconate Titanate thin film while still operating the device in an ambient environment at Complementary-Metal–Oxide–Semiconductor compatible voltages. By electrically detuning the operation point along the nonlinear regime of the resonator, the number of electrical and light-matter interaction signals generated based on higher-order non-Eigen modes can be controlled meticulously. This tunable multichannel generation enabled microdevice is a potential candidate for a wide variety of applications ranging from Radio Frequency communication to quantum photonics with an attractive MEMS-photonics monolithic integration ability.

Nonlinear phenomena in optics such as frequency comb generation^1^, parametric oscillation^2^, and higher-order harmonic generation^3–5^ (HHG) have been actively investigated because of their strong significance in multiple sectors of science and engineering such as spectroscopy^6^, telecommunications^7^, quantum information processing^8^, etc. The multitasking feature of a single device that generates multiple coherent signals will lead to shrinkage of the system footprint. However, most of the HHG generation schemes involve complex and expensive experimental setup that poses a bottleneck for portable and cost-effective System-on-Chip (SoC) implementation. Additionally, once the photonics module is fabricated, the options for real-time control of the number of harmonics generated are scarce. An electromechanical analogy of the abovementioned nonlinear optical phenomena has been demonstrated on a micro-scale in capacitive^9,10^ and piezoelectric^11,12^ Microelectromechanical System (MEMS) resonators. Frequency comb^13–15^ and HHG^16^ demonstration are proof of the “single device, multiple frequency” concept. Nonlinear behavior in MEMS undoubtedly paves way for MEMS-based applications such as electromechanical shift registers^17^, logical gates^18^, miniaturized resonant oscillators^19^, sensors^20^, phonon computing^21^, etc. In capacitive MEMS, the internal resonance phenomenon^22^ is utilized to couple energy from fundamental mode to higher-order Eigenmode. Nevertheless, only a low count of HHG has been realized so far due to low coupling efficiency. Piezoelectric transduction^23^ on the contrary has an upper hand over other energy coupling methodologies as it has a higher coupling efficiency, which leads to the generation of a large number of harmonics for a lower actuation energy level. Gallium Arsenide (GaAs) resonators^24^ based systems are an example of nonlinear physics-based applications. GaAs resonators have showcased promising multimode applications where harmonic oscillation modes were exploited to attain Boolean operators^25^, which will be a cornerstone for the futuristic MEMS-based computers^26^. But despite a low actuation voltage requirement, GaAs resonators need extremely low temperatures (< 4 K) and a very high vacuum (~ 2 × 10^−7^ mBar) environment^27^ to harness their nonlinear features. These critical operation conditions are challenging to implement on a portable chip-scale device. Hence an alternative scheme has to be conceived to realize an all MEMS computational unit which can not only generate a large number of modes using a low actuation power but at the same time also work in an ambient environment.

Each higher-order harmonic signal generated by MEMS corresponds to a unique mechanical vibration pattern. This implies that the micro-resonator that generates N-modes can modulate an incoming stream of photons in N-unique patterns. This concept unveils the possibility of the realization of a Single Input Multiple-channel Output (SIMO) Acousto-Optic Modulator (AOM). AOMs are widely used in optical systems and are based on the photon-phonon interactions between the incident light and the motion of the modulating medium. Traditionally, Surface Acoustic Wave^28,29^ (SAW) based MEMS devices are used to launch an acoustic wave of a particular frequency or a set of frequency (depending on the range of the drive signal) such that the periodic displacement of propagated wave modulates the refractive index of the incident light. Recent fabrication advancements have allowed the encapsulation of optical waveguide on the resonator itself^30^, and bulk modes are being investigated to implement high performance AOM^31^. However, these modulators largely can operate only at a single frequency for a single driving frequency i.e., SISO and due to this, only one channel modulation can be attained. This shortcoming limits the optical signal processors from efficiently using their parallel processing capability.

Here, we experimentally realize an N-channel AOM and frequency synthesizer using a single mechanically suspended Lead Zirconate Titanate (PZT) micro-resonator by electrically driving the fundamental resonance mode into strong nonlinearity such that non-Eigen modes at harmonic frequencies are excited. Soft PZT is used in this work to achieve a higher degree of nonlinearity ^32^. Dielectric and piezoelectric nonlinearity are the main phenomena through which the harmonics are generated. When the amplitude of the pure sinusoidal time dependent drive signal exceeds the threshold level, higher-order displacement signals and voltages are observed^33^. These highly efficient multi-domain HHG (N > 100) demonstrations are carried out by operating the MEMS resonator at ambient atmospheric pressure at complementary metal–oxide–semiconductor (CMOS) compatible voltage levels at room temperature. Unlike traditional approaches where N-frequency-selective tanks are required for N-oscillations, here we achieve N-harmonic oscillation signals using a single resonator. Most importantly, we demonstrate the new SIMO ability of thin-film piezoelectric AOM. Using an HHG-MEMS, the incoming laser beam will be modulated not only at the fundamental resonance mode of the resonator but also at all the N-harmonic modes, which will result in an N-channel spatial modulation. In addition to the generation of new non-Eigen modes, the harmonic count can be regulated using a voltage signal. This experimental validation of a multichannel AOM and frequency synthesizer functionality using a single MEMS micro-resonator is a stepping stone towards the realization of the MEMS-optics integrated system.

## Results

### Design concept and application

**Figure 1:**
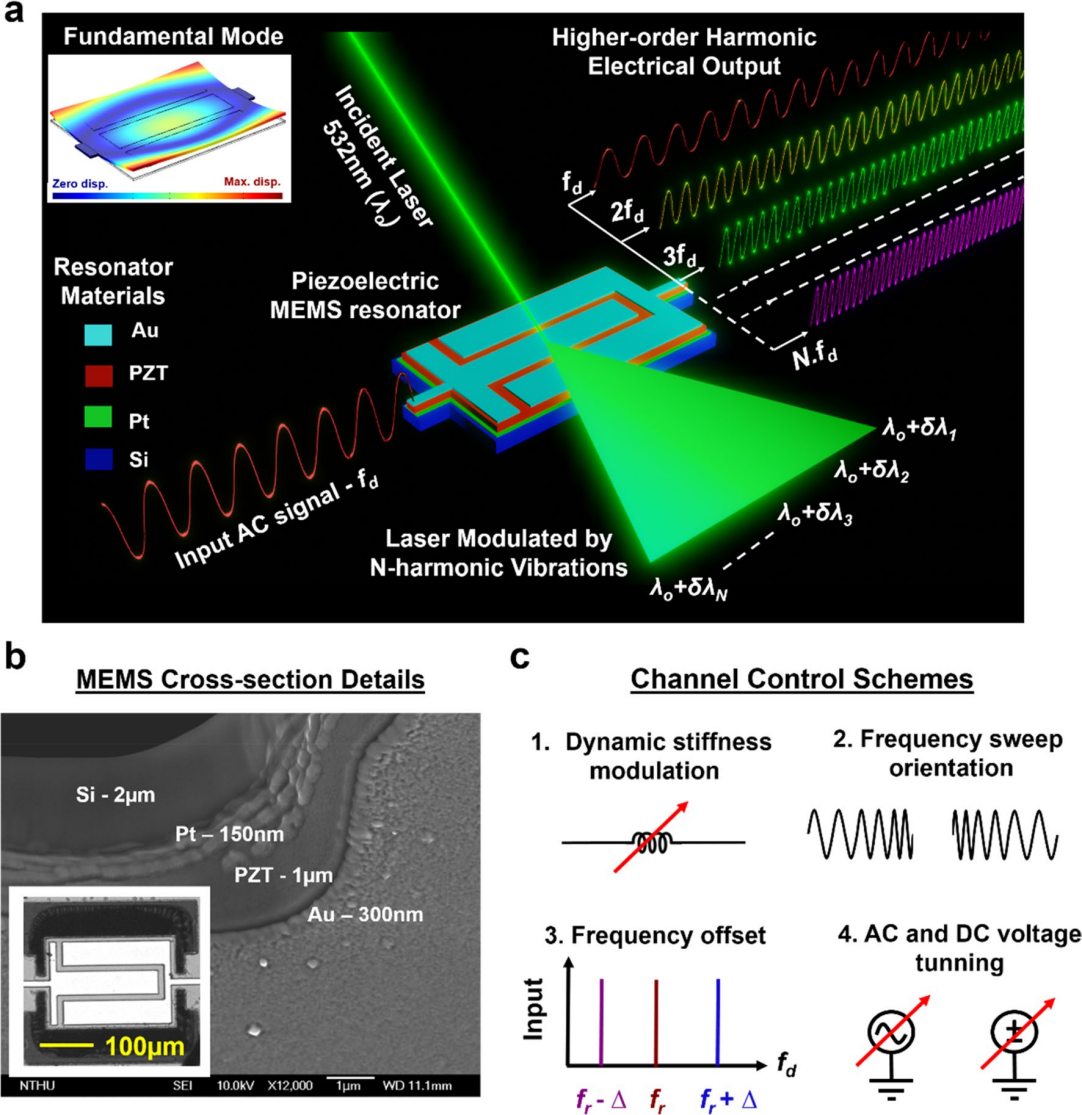
Nonlinear MEMS resonator-based multi-domain (Opto-Electro-Mechanical) signal processor. (**a**) Schematic of the proposed chip-scale low powered MEMS-based SIMO AOM and frequency synthesizer operating at CMOS compatible voltages in the ambient environment. For the AOM implementation, an AC electrical signal is provided to the piezoelectric device and a laser is focused on the resonator surface. The backscattered laser signal will be modulated as a function of the mechanical vibration and nonlinearity of the resonator, thereby providing a range of optical modulation options. Likewise, for a frequency synthesizer application, an AC electrical signal drives the device into tailored nonlinearity, and the harmonics of the drive signal are observed at the output electrode. The inset shows the fundamental mode shape of the resonator. (**b**) Scanning Electron Microscopy (SEM) image of the resonator cross-section with the thickness details of each thin-film material. The inset shows the Optical Microscopy (OM) image of the resonator. (**c**) Different channel control schemes that can be implemented using a complete electrical interface. The number of harmonics are befittingly controlled by engineering the resonator operation point along the hysteresis curve of the nonlinear resonator.

In this work, a thin film PZT-on-Silicon (TPoS) based MEMS resonator serves as the source for uniformly spaced frequency tones, and it has been employed to implement chip-scale multichannel signal processors in optical and electrical disciplines. As HHG is the underlying requirement for both multichannel AOM and frequency synthesizer applications, a single-tone drive signal (*f*_*d*_) close to the desired resonant frequency (*f*_*r*_) is provided to the resonator’s input electrode with an amplitude high enough to drive the device into nonlinearity as shown in Fig. 1a. Upon driving the resonator in an engineered nonlinear configuration, integral multiples of *f*_*d*_ with good Signal-to-Noise Ratio (SNR) are observed. Owing to the bias dependent domain wall motion of PZT^34,35^ and optimal resonator design featuring multi-degree freedom, a large number of harmonics can be generated by solely injecting a low amplitude single tone AC voltage into the resonator. To readily achieve nonlinear operation, the mode shape of the fundamental resonance mode is critical as it dictates the dynamic spring constant at the frequency of interest. Flexural vibration such as flapping mode as shown in the inset of Fig. 1a possesses large structural displacement and has been widely considered as a category of deformation which features lower power handling ability^36^. This attribute permits the resonator to be driven into nonlinearity using very low actuation voltage. The resonator stack details along with the OM image of the device is presented in Fig. 1b. The resonator is a rectangular shaped suspended structure with two anchors to support the device and enable electrical signal routing to the resonator’s active area as shown in Fig. 1b. When an input AC signal is delivered via the input electrode, due to the non-centrosymmetry of PZT an equivalent strain is generated. As a result, the electrical signal is converted to the mechanical displacement of the suspended structure through the converse piezoelectric effect of PZT thin film. The motion of the resonator is amplified when *f*_*d*_ matches *f*_*r*_ of the device. Consequently, through the direct piezoelectric effect of PZT the charge generated by the motion of the plate resonator is detected by the output top electrode.

Two distinct applications are demonstrated here to emphasize the wide spectrum of possibilities of MEMS-based controllable HHG concept. Figure 1a dictates the SIMO configuration of both AOM and a frequency synthesizer. The AOM concept is validated using a 532 nm laser incident on the suspended resonator structure. The resonator is driven into a nonlinear regime using a single tone AC signal, and in the linear zone modulation occurs only at *f*_*d*_. However, when AC amplitude is increased, the resonator enters the nonlinear regime and it vibrates at the integral multiples of *f*_*d*_. The displacement corresponding to each vibration mode will modulate the frequency and phase of the incident light uniquely. Due to N modes generation, the monotonic laser will be modulated by the resonator velocity corresponding to each harmonic frequencies of *f*_*d*_. This is a real-time demonstration of SIMO AOM using the light-matter interaction feature of the vibrating thin film and laser. A frequency synthesizer is an example of a fully electrically interfaced SIMO system, where the traditional high power consuming integral-frequency scaling block is eliminated as the nonlinear MEMS itself serves as the harmonic generator. The electrical signal extracted from the output electrode will contain equally spaced spectral tones in the multiples of *f*_*d*_. This resonator can be configured as an N-oscillation system in the closed-loop arrangement such that N oscillators can be realized using just one resonator. Such one-resonator N-oscillation systems will find a strong footing in Internet-of-Things (IoT) where multiple devices communicate at different frequencies. Modern technologies demand simultaneous information transfer between different information hubs such as environmental sensors, telecommunication centers, health monitoring applications, etc. For an efficient data transmission/reception, a high performance timing reference is quintessential as synchronization between different channels has to be maintained. The data transfer of each unit is controlled by its clock, and in a system, each sensor operates and transmits/receives data at its unique frequency. Thus for an N-functional unit, N-timing references would be needed, and this would increase the system footprint and also the power consumption. Although an array of frequency selective tanks can be fabricated lithographically, the frequency variations between each resonator may still exist due to fabrication variations. In this work, the system configuration is simplified not only in terms of power consumption but also in packaging as this PZT-based SIMO can be realized in ambient conditions at CMOS compatible voltages.

For a system that can generate harmonics, it is critical to devise methods to control the number of output tones. By tuning the point of operation along the nonlinear regime, the number of harmonics generated at the output can be controlled. As shown in Fig. 1c, we demonstrate four methods to program the output pattern by merely varying one or a combination of these options which will be detailed in the coming sections. Two different resonator samples based on the same mode shape but different operation frequencies are investigated in this work (Fig. 6c).

### Nonlinear MEMS characterization

**Figure 2:**
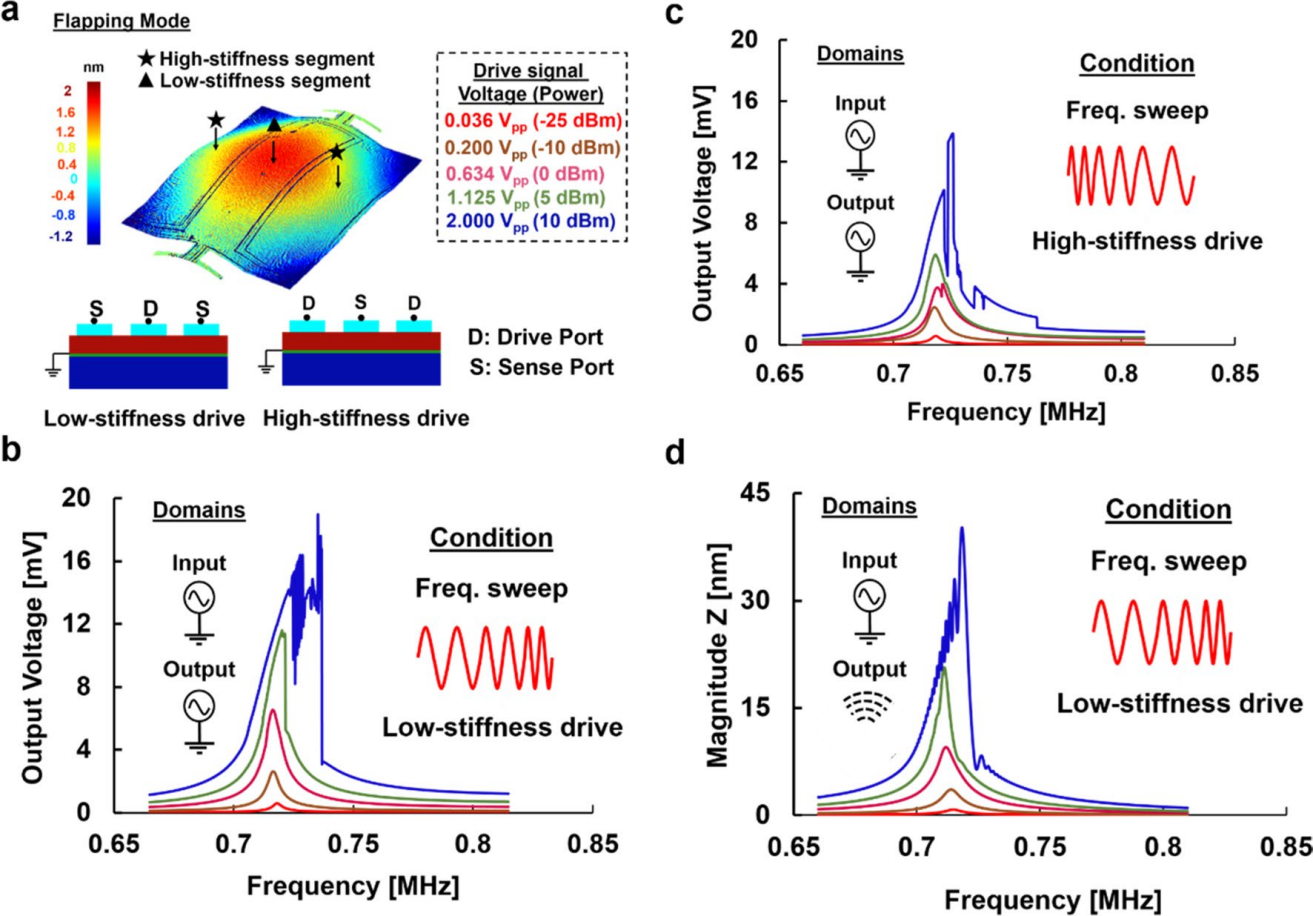
Electrical and mechanical frequency spectra of Device A. (**a**) The mode shape of the resonator with different stiffness regions labeled. Drive/sense configurations employed for nonlinearity measurement of the resonator are also shown. The inset presents the voltage to power equivalency for all measurements in this work. (**b**) Forward frequency sweep for a low-stiffness drive. (**c**) Reverse frequency sweep for a high-stiffness drive. (**d**) Laser Doppler Vibrometer (LDV) out-of-plane motion measurement for forward frequency sweep in the low-stiffness configuration.

The measured vibration pattern of the flapping mode and different drive/sense setups used to tune the nonlinear behavior of the resonator over a range of applied input voltages (and equivalent power) are shown in Fig. 2a. At resonance, the average displacement is higher at the central portion when compared to the sides and hence when the center electrode is used to drive the resonator it is termed as low-stiffness drive, and high-stiffness drive when the side electrodes are used for actuation. A Zurich HF2LI Lock-in amplifier is used to drive and sense the resonator’s electrical response (Fig. 7b)*.* Due to a strong hardening feature, the resonator tends to go into a more nonlinear regime when the frequency is swept from low to high frequency. Driving a resonator in the low-stiffness region pushes the device into more nonlinearity than operating in the high-stiffness region as the effective stiffness for the former configuration is lesser than the later^37^. As seen in Fig. 2b-c the resonant peak of Device A for the 36mV_pp_ drive is consistent for all the drive configurations as it is in the linear region of operation. However, when the input is increased gradually, the nature of response for each of the configurations varies corresponding to varied nonlinear behaviors. Forward frequency sweep (low to high frequency) in a low-stiffness drive configuration shown in Fig. 2b has the highest output amplitude with maximum nonlinearity whereas a degenerate nonlinearity can be observed in Fig. 2c with the reverse frequency sweep in a high-stiffness drive configuration. To confirm this Duffing nature, mechanical vibration response is monitored using a Laser Doppler Vibrometer (Fig. 7c) for the maximum nonlinear configuration as shown in Fig. 2d and the out-of-plane displacement was recorded. Doppler effect is utilized to attain the displacement information of the resonator surface. A power sweep for the forward frequency sweep was conducted and the displacement nature matches very closely with the electrical readout trend of Fig. 2b. This shows that the mechanical displacement of the microresonator is also modulated concurrently by the driving electrical signal and the device’s stiffness configuration.

### SIMO acousto-optic modulator

**Figure 3:**
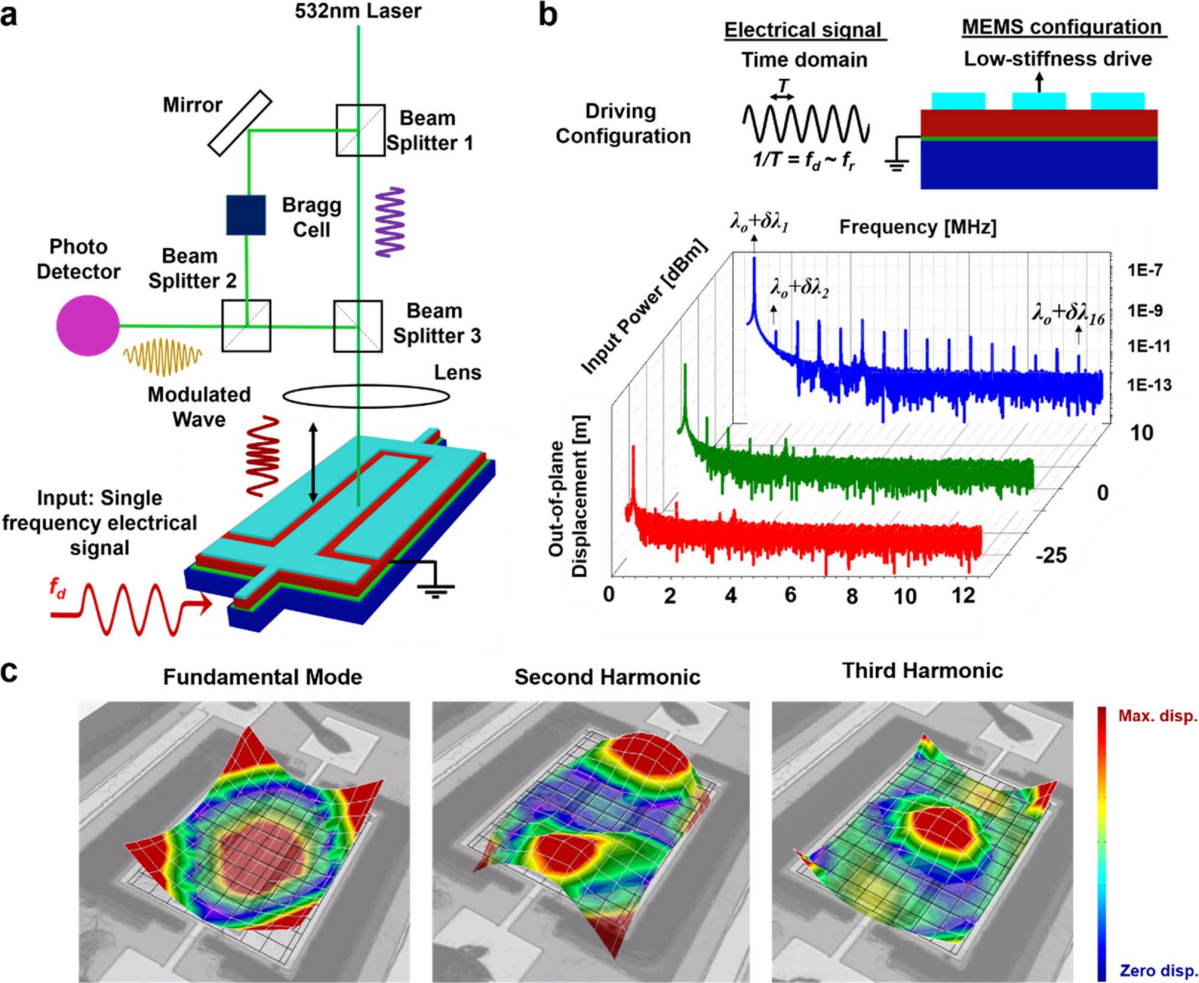
SIMO Acousto-Optic Modulator. (**a**) Schematic of the SIMO AOM measurement setup. The source laser beam is split by beam splitter 1 into a measurement beam and a reference beam. The measurement beam passes through beam splitter 3 and travels to the MEMS resonator surface from where it gets reflected back. This modulated wave is then merged with the reference beam at beam splitter 2 onto the photodetector. The superimposed interference pattern contains the vector details of the displacement/velocity of the vibrating surface. The change in the optical path length per unit time is a function of the Doppler frequency shift of the reflected laser beam. (**b**) The device and electrical signal drive configuration for the demonstration of the light-matter interaction across different frequencies. The amplitude of the single tone AC signal is varied to control the extent of modulation. Strong harmonics are observed when MEMS operates in a highly nonlinear region. (**c**) Out-of-plane vibration pattern of the fundamental, second, and third harmonic modes. The first mode is an Eigenmode whilst the second and third are non-Eigen modes that are generated due to the nonlinearity of MEMS.

An experimental demonstration of the SIMO AOM is performed using one monochromatic laser source and a single frequency AC signal input. The electrical signal actuates the resonator (Device A), and the nonlinearity level of the device is controlled by tuning the drive configuration and signal amplitude. For this experiment, to leverage the nonlinear features constructively the driving frequency is chosen such that it is in the close vicinity of the flapping mode resonance frequency. The path of the impingent laser signal and the interferometry setup that uses the Doppler Effect to extract the frequency and phase information from the backscattered modulated laser beam is shown in Fig. 3a. The laser will be modulated in frequency and phase by multiple acoustic waves simultaneously as the nonlinear MEMS generates N-harmonic output at a given point in time. The modulated wave is superimposed with the reference beam on the photodetector, which is then used by the system software to perform demodulation. It can be seen from Fig. 3b that increasing the drive signal strength enhances the HHG phenomenon, and this is due to the dominance of the extrinsic nonlinearity of PZT thin film. For a drive voltage of 2V_pp_, the HHG is very strong such that for an output frequency span of 0.5–12.5 MHz all the 16 harmonics can be observed as seen in Fig. 3b. It implies that the incoming photon stream is modulated at 16 different frequencies concurrently as a result of the strong light-matter interaction occurrence at the center of the MEMS resonator. This phenomenon paves a path for the realization of uncomplicated multichannel AOM that can operate efficiently even in ambient conditions using a singular low powered electrical drive signal and a photon source. It is important to notice that N = 2 to 16 harmonic modes are non-Eigen modes as these modes do not exist in the linear system (Supplementary Fig. S3) because of which it is challenging at this point of time to extract their mode shapes analytically or in Finite Element Analysis. A two-dimensional scanning of the resonator is performed to garner the vibration magnitude and phase details experimentally. Spatial data reconstruction is performed to visualize the mode shapes as shown in Fig. 3c. The successful demonstration of a low frequency (kHz-MHz) vibration modulating a Terahertz Electromagnetic (EM)^38^ signals can be used to realize low powered sub-millimeter wave modulators and sensors. This concept can be extended by integrating an optical waveguide directly on the resonator such that the EM signal will be modulated uniquely at different harmonic frequencies.

### SIMO frequency synthesizer

**Figure 4:**
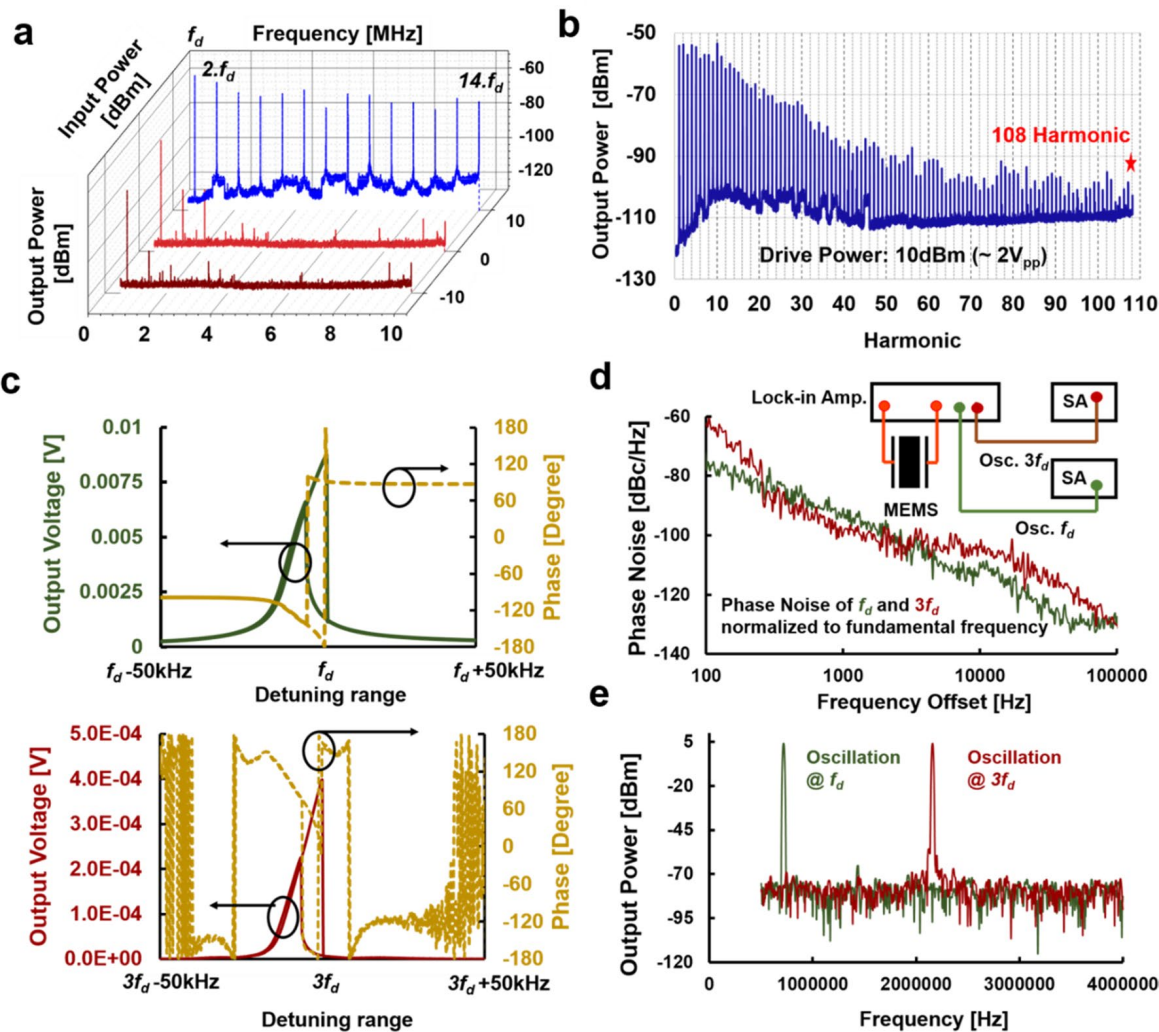
SIMO Frequency Synthesizer. (**a**) Output spectra of HHG for a single tone frequency input. The SA BW is set to 11 Hz for a better SNR. The number and strength of the harmonics are enhanced when the drive power is increased. (**b**) The electrical output spectrum for Device B when the resonator is driven at a frequency corresponding to the maximum range of nonlinearity. Reduced dynamic stiffness and increased extrinsic nonlinearity aid the generation of very strong HHG. (**c**) The frequency response of the resonator across the fundamental and third harmonic frequency when the AC input signal is swept bi-directionally along the fundamental resonant mode. (**d**) Demonstration of frequency synthesizer using a single MEMS resonator without any multiplication block. An oscillator operates at both *f*_*d*_ and 3*f*_*d*_ concurrently. The inset shows the measurement setup. (**e**) Oscillation spectra of two loops operating at *f*_*d*_ and 3*f*_*d*_, displaying the SIMO capability of PZT based nonlinear MEMS.

Here, we demonstrate a single device serving as the frequency generator for N-different applications which can potentially lead to the scaling down of system footprint and power consumption. A fully electrically interfaced N-harmonic generator is implemented by driving the resonator (Device A) at a highly nonlinear operation point in the low stiffness configuration as shown in Fig. 4a. For this measurement as well the single tone drive frequency is selected close to *f*_*r*_. The output electrical signal is connected to the spectrum analyzer (SA) to investigate the harmonic frequencies. Equally spaced power outputs can be observed and drive level-dependent harmonic generation can be seen in the electrical domain as well. To further extend the HHG count, the MEMS geometry is altered to attain a lower dynamic stiffness for similar driving power. Device B is designed to have its flapping mode resonance at a lower frequency (*f*_*r*_ ~ 320 kHz). At a reduced operational frequency, owing to a lower dynamic stiffness the resonator will exhibit a stronger nonlinearity than Device A. When being operated at *f*_*d*_ around resonance for an input power of 10dBm with the device configured in a highly nonlinear arrangement, a train of harmonics can be observed for an SA output frequency span of 0.1-35 MHz as shown in Fig. 4b, with the highest harmonic count being 108. This work reports for the first time a record-breaking number of harmonic counts in an experimental demonstration of HHG. To utilize these harmonic signals in Radio Frequency communications a frequency synthesizer is realized by implementing oscillators operating at integral frequencies of the drive AC signal. An N-oscillator module is demonstrated using the Multi-Frequency (MF) and Phase-Locked Loop (PLL) feature of the Zurich HF2LI lock-in amplifier. Using the MF feature it is possible to observe the output voltage magnitude and phase transition details of the fundamental and harmonic frequencies simultaneously. Firstly, the input AC signal is swept along the flapping mode resonance frequency to initiate the nonlinear harmonic generation process. The output electrical signal collected from the sensing electrode is analyzed across different harmonic frequencies as shown in Fig. 4c. To realize the closed-loop oscillator system, two different oscillators simultaneously operating at *f*_*d*_, and 3*f*_*d*_ are implemented as shown in Fig. 4d-e. This measurement validates the feasibility of the elimination of the power-hungry frequency multiplier block used in the traditional frequency synthesizer system.

### Fully electrically interfaced programmable HHG

**Figure 5:**
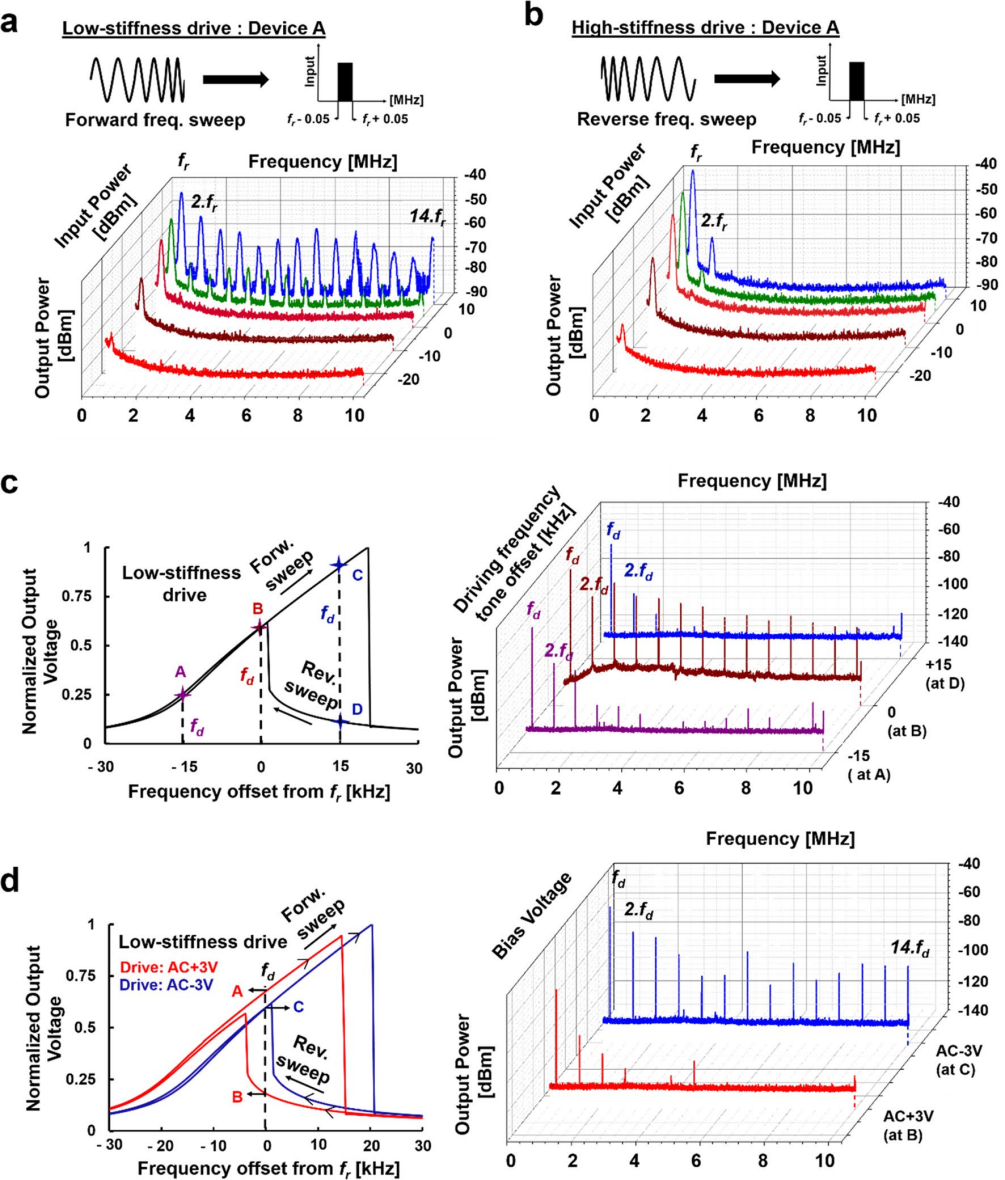
Fully electrically interfaced HHG control schemes. (**a**) and (**b**) are the output spectra for Device A operating in the high and degenerate nonlinear regime respectively for an input frequency sweep of *f*_*r*_ ± 50 kHz. For this HHG study, the input AC signal sweep has a definite bandwidth and is not a single tone. (**c**) The output voltage plot for a bidirectional frequency sweep of the resonator along *f*_*r*_ ± 30 kHz is presented and output voltages corresponding to three frequencies are identified. For the single tone based HHG control, when the device is driven at each of these frequencies different harmonic outputs are attained. The count and the strength of the harmonics directly correspond to the output voltage at *f*_*d*_. (**d**) The output voltage plot for a bidirectional frequency sweep of the resonator along *f*_*r*_ ± 30 kHz is presented for different DC bias voltage. The frequency shift is DC voltage polarity dependent; hence for different DC bias voltages, different counts of harmonics can be achieved for the same *f*_*d*_.

Figure 5a shows the highly nonlinear drive/sense device configuration operated in a forward frequency sweep of ± 50 kHz around the resonant frequency. For an input of -25dBm, only the fundamental mode can be observed which is indicative of the fact that there is no HHG phenomenon and that the resonator is still operating in the linear region. As the drive amplitude is increased, the HHG phenomenon starts unveiling, and at 0dBm the 2^nd^ harmonic peak is observed. For a 10dBm drive, the HHG is very strong such that for an output frequency span of 0.5-10 MHz all the 14 harmonics can be observed. To investigate the ability to control the harmonic generation by altering the drive/sense scheme, the resonator is driven in the high-stiffness configuration with a reverse frequency sweep as shown in Fig. 5b. Since the mode undergoes hardening, reverse frequency sweep will yield a lower nonlinear frequency response. Corroborating the conclusions arrived in Fig. 2b-c, the HHG counts are lesser in this condition as can be seen in Fig. 5b. This measurement proves the strong digitally controllable harmonic feature of a MEMS-based HHG setup. Merely swapping the drive terminals and the orientation of the frequency sweep can suppress or generate harmonics to cater to the varying demands of multiplexing applications.

In addition to the stiffness modulation and the sweep orientation approaches, the operation point of a resonator along the hysteresis curve can be tailored by (i) detuning the drive frequency offset, and (ii) applying an external DC bias voltage. The idea for this approach is derived from the fact that the count of the harmonic generation corresponds to the output amplitude of the frequency response. When the device is driven into nonlinearity, it results in an interesting frequency behavior where the device has more than one output amplitude at a given frequency due to hysteresis as shown in the bi-directional frequency sweep response of Fig. 5c-d. If the resonator is forced to operate in that particular bandwidth, the device voluntarily operates at a lower energy state as it is for any system when faced with multiple energy levels. As a result, in such cases, the device operates along the lower amplitude branch. This feature is exploited meticulously to attain a tailored number of HHG and for this, we use the techniques mentioned above. Here these schemes of external tuning are demonstrated while the resonator is configured in a highly nonlinear regime for a constant input power of 10dBm. For the first tuning scheme, multiple drive frequency tones that map to different output amplitudes were chosen as shown in Fig. 5c. The output spectrum trace corresponding to the *f*_*d*_ that has the maximum output voltage shows the strongest HHG phenomenon. At *f*_*r*_ + 15 kHz, the bidirectional frequency sweep of the suspended resonator yields two amplitudes for the same frequency. Hence, when driven at a constant frequency of *f*_*r*_ + 15 kHz, the device operates at point D and yields a lower HHG count than *f*_*r*_-15 kHz as the former maps to a lower amplitude than the latter. DC bias voltage tuning^39^ of the resonant frequency is next explored to change the point of operation in the nonlinear regime while maintaining the same *f*_*d*_. This is based on the inherent polarization reversal mechanism of materials such as PZT^40^. This unique feature of the material provides yet another degree of freedom in this HHG study. Switching the polarity of the bias voltage generates an offset in the frequency response as illustrated in Fig. 5d. Based on this observation, if the operating frequency and device configuration are predetermined, then the number of harmonics will eventually then be modulated by the bias dependent output amplitude. Hence building upon this concept, depending on the requirement of the harmonic counts at the output, suitable biasing can be applied to switch the operation point between the upper and lower branches as shown in Fig. 5d. When the polarity of the DC bias was changed from -3 V to + 3 V, the entire frequency response shifts resulting in *f*_*d*_ to fall in the hysteresis region with dual amplitude (Points A and B). As explained earlier, the resonator will operate at point B as it is a lower energy state and thereby yielding a lower harmonic count at the output.

## Discussion

The digitally programmable SIMO AOM and frequency synthesizer experimental results illustrated in this work using PZT thin film demonstrate devices working in the sub-MHz range that successfully caters modulation and reference signal purposes respectively for multiple applications in the optical and electrical disciplines concurrently. This approach overcomes the hurdles such as expensive and bulky setups, high vacuum requirement, non-IC compatible DC voltages, weak electromechanical coupling, etc. which are common in the conventional techniques. Using the piezoelectric thin film based resonators, operation at other frequencies within the range where material nonlinearity is significant is also possible. We have observed a strong HHG in different resonator designs fabricated in the same batch operating at different fundamental modes even in the MHz range. To further extend this study, it is feasible to alter the resonator’s material configuration, and the nonlinearity window of operation can be tailored as well by choosing a different primary mode of operation as the resonator’s dynamic behavior varies with the target resonant mode. Our work using PZT with its excellent electromechanical coupling coefficient supersedes not only in the quality and control of the harmonics but also attains a good SNR for its harmonics as can be seen in Fig. 4 and 5.

Albeit a highly efficient HHG system has been explored in detail here, there are many options still unexplored that can improve the nonlinear physics-based system performance. A nonlinear frequency response similar to Fig. 2b has been observed in nanoelectromechanical resonators^41^; however, an HHG aspect wasn’t explored by the authors. Hence, we strongly believe that HHG study can be extended to nanoscale resonators as well which would open up not only new engineering aspects but also unveil new dynamics in mechanical, electrical, optical, and material science and engineering domains. Well established piezoelectric materials with strong coupling features such as Aluminum Nitride, Lithium Niobate, etc. can be explored for extending the range of operation to an even higher frequency domain (MHz-GHz). Aluminum Nitride based HHG system will enable monolithic integration of CMOS and MEMS^42^ on a single chip which can cater to the ever-growing demands of the Internet-of-Things. In the optical domain, acoustic modulators have been used to perform wave mixing across a range of frequencies. Hence, a MEMS-based reconfigurable AOM can not only produce harmonic offset and phase modulation for the impingent monochromatic laser but also provide a wide spectrum of mixing options based on light-matter interaction by injecting the resonator with multiple drive signals strong enough to drive the targeted Eigenmodes into nonlinearity. The possibility of integrating optical signal paths on the suspended MEMS resonator can further shrink down the optical modulator setup leading the way to multiport systems.

In conclusion, we have successfully demonstrated the idea of a digitally controllable MEMS-based SIMO AOM and frequency synthesizer that can provide up to second-order overtones with a single AC signal when the resonator is operated at room temperature under atmospheric pressure condition. This concept is projected to have a strong footing in both electrical and optical communication domains as the MEMS resonator can serve as a signal processor for multi-frequency (or wavelength) applications simultaneously. Several fully electrically interfaced modulation techniques that can precisely control the nature of harmonics have been verified. Moreover, as a single signal is responsible for the generation of equally spaced spectral signals, it is anticipated that the stability of the array of tones can be well controlled by merely stabilizing the fundamental tone frequency. The extremely low power consumption (< 10mW) of the system to generate harmonics is yet another notable feature of this PZT based HHG system. The extrinsic nonlinearity of PZT and the meticulous design of the interdigitated two-port resonator were utilized to engineer the electromechanical coupling efficiency and the dynamic stiffness. The interdisciplinary research results demonstrated here set a landmark in the field of HHG studies across multiple domains which have been actively investigated globally to translate the nonlinearity of material and/or structure to applications such as electrical and optical signal modulation modules, RF communication, multiplexing systems, logic operators, quantum computing, phonon manipulation, etc.

## Methods

### Microresonator fabrication

**Figure 6:**
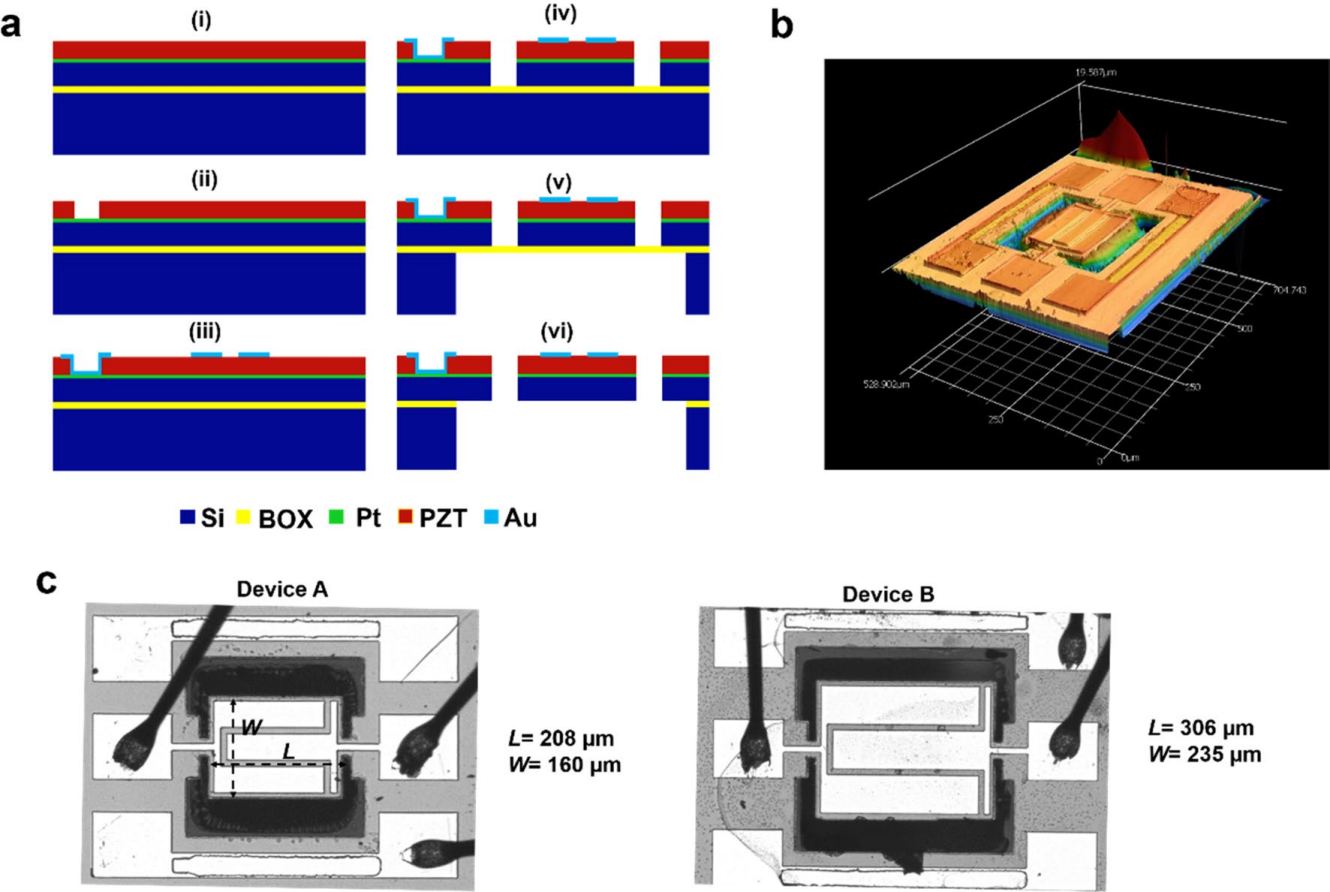
Device fabrication. (**a**) Fabrication flow of the PZT TPoS MEMS resonator. (i) Incoming PZT thin film on SOI wafer. (ii) First mask: Pattern PZT. (iii) Second mask: Pattern top electrode. (iv) Third mask: Pattern PZT/Pt/SCS for resonator geometry definition. (v) Fourth mask: Backside etch for device release. (vi) Dry etch to remove the BOX. (**b**) Profile of Device A measured using a Keyence laser microscope under static condition. (**c**) Optical microscope images of Devices A and B with their dimensions labeled.

The resonators were fabricated using a four-mask PZT Silicon-On-Insulator (SOI) microfabrication process provided by GlobalMEMS Co. Ltd as shown in Fig. 6a. The process starts with a bare SOI wafer (Active-Si: 2 µm/ Buried Oxide: 0.5 µm/ Handle-Si: 400 µm). Over the 2 µm thick silicon, 150 nm platinum is deposited. Platinum serves as the bottom electrode for the resonators. A 1 µm PZT thin film is then deposited by the standard sol–gel process and then patterned to facilitate a through via to enable top and bottom electrode contact. A 300 nm thick gold is sputtered over PZT and patterned using a lift-off process. Gold electrodes function as the input signal supply and output electrical signal sensing medium for the devices and they are routed suitably for probing pad interconnections purposes. The device geometry is defined using a dry etching process known as Reactive Ion Etching (RIE). Next on, backside Deep RIE is used to etch the 400 µm thick Handle- Si. The Buried Oxide (BOX) serves as the etch stop layer for both the front and backside etching. Finally, dry etching of BOX thin film is performed to release the device. The laser microscope image that maps the device profile under static conditions is shown in Fig. 6b.

### Individual device details

Different structural geometry and drive/sense signal configurations were explored to investigate the HHG phenomenon in MEMS TPoS resonators. The devices’ optical images are presented in Fig. 6c and their details are stated below:

#### Device A: *f*_*r*_ ~ 715 kHz

The fundamental flapping mode resonant frequency for Design A is roughly around 715 kHz and the rectangular resonator has released device dimensions of 208 µm × 160 µm. The device’s resonant frequency may vary slightly from sample to sample because of the fabrication variation across the wafer. Asymmetric electrode design is used for the desired flapping mode i.e., the electrode coverages of drive/sense region over the active resonator structure are not equal.

#### Device B: *f*_*r*_ ~ 320 kHz

A larger version of Design A was designed to extend the nonlinearity hysteresis loop. The main resonant body dimensions are 306 µm × 235 µm. The drive/sense and ground electrodes are also scaled up accordingly to ensure enhanced transduction. Albeit the same *L*/*W* ratio as Design A was maintained, the effective stiffness is expected to be lesser for Device B. The fundamental flapping mode occurs at a much lower drive frequency than Device A.

### Measurement setup

#### Electrical input—electrical output

**Figure 7:**
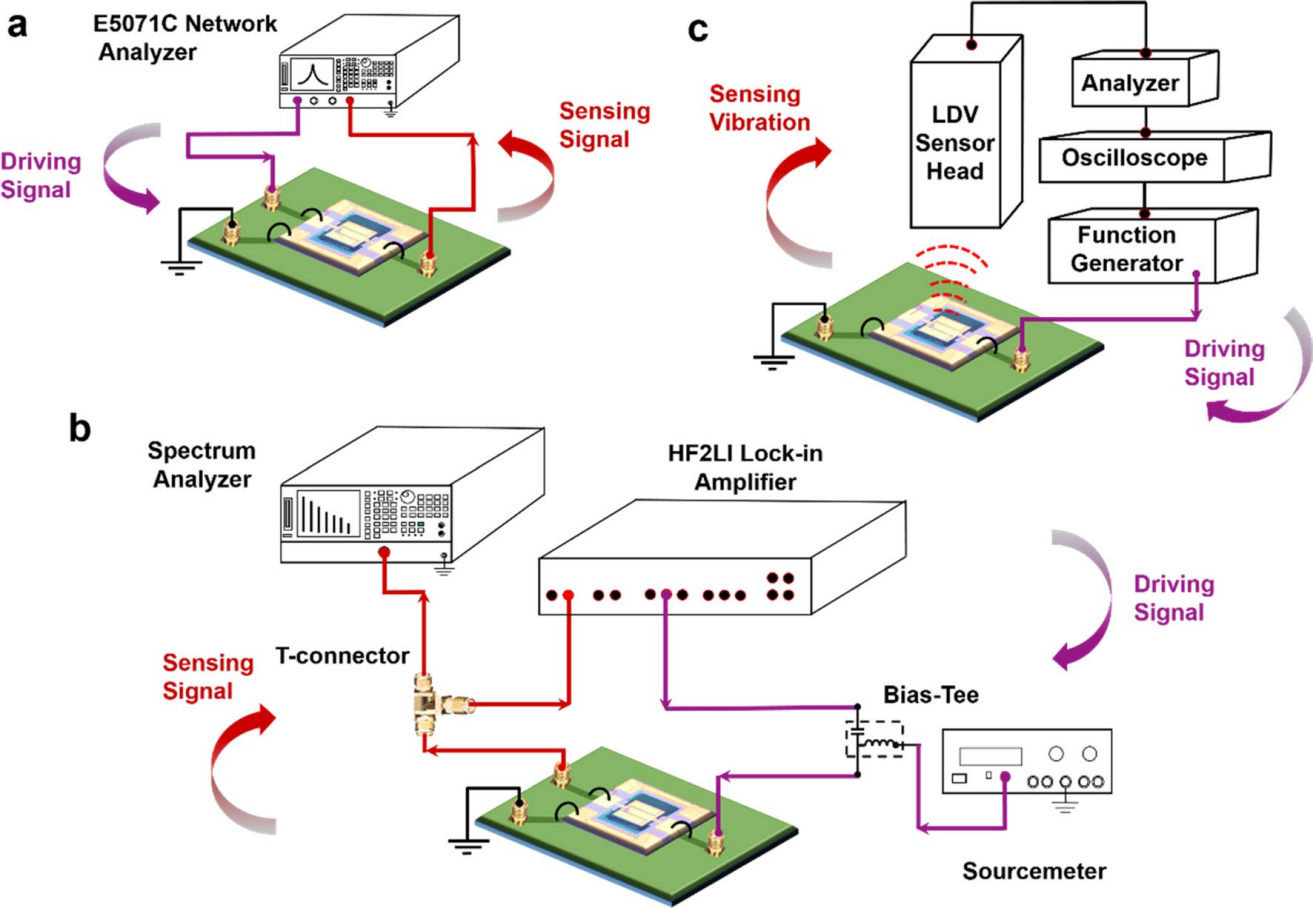
Measurement setup. Electrical-in Electrical-out arrangement: (**a**) Agilent Network Analyzer serves as the time-varying signal source. The driving power and the sweep frequency range can be precisely controlled using the Network Analyzer. (**b**) Zurich Instruments’ HF2LI Lock-in amplifier’s output is connected to the input of the resonator and the resonator’s output is connected back to the Lock-in amplifier and also to the SA using a T-connector. SA is used to record the harmonic generation for different drive conditions of the device. (**c**) Electrical-in Mechanical-out arrangement: An internal signal source of the LDV drives the resonator into resonance and the out-of-plane displacement of the resonator is recorded by the sensor head. Information in both the time and frequency domain can be attained. In addition to the magnitude and phase details, graphical files of the mode shape vibration are also generated.

A complete electrical interface measurement setup was used to investigate the resonant behavior of the TPoS MEMS device. Figure 7a shows the scheme where the device is operated using Agilent Network Analyzer E5071C. Network Analyzer (NA) is deployed to carry out initial resonator characterization and to extract the parameters relevant to the mode of interest (Supplementary Fig. 2 and 3). AC power is supplied to the device along with the desired frequency range and the transmission plot is recorded in the NA. Next on, Zurich Instruments’ HF2LI Lock-in was used as the signal source to perform bidirectional frequency sweep and also for the harmonic generation studies. The schematic for the aforementioned case is shown in Fig. 7b. Keithley Sourcemeter was used to tune the DC bias potential of the resonator. To observe the harmonics in the frequency domain, the output of the resonator is connected to the Spectrum Analyzer.

#### Electrical input—mechanical output

Laser Doppler Vibrometer based non-contact vibration measurements were carried out to study the structural dependent nonlinearity and HHG features. An electrical AC driving signal was provided to the resonator’s top electrode using the system’s internal function generator. The Polytec MSA-100-3D LDV arrangement is based on the concept of the Doppler Effect i.e., sensing the frequency shift of backscattered light from a moving surface. Displacement magnitude and phase information of the fundamental mode and also higher harmonic modes for different driving power levels, varied frequency sweep patterns, etc. were garnered using the setup shown in Fig. 7c.

## Supplementary Information

Supplementary Video 1.

Supplementary Video 2.

Supplementary Video 3.

Supplementary Information 1.

## Data Availability

All data are available in the main text or the supplementary materials.
