# Supplementary material for: Controllable multichannel acousto-optic modulator and frequency synthesizer enabled by nonlinear MEMS resonator
Source: Sci Rep. 2021 May 25;11:10898. doi: 10.1038/s41598-021-90248-w (PMC8149383; doi:10.1038/s41598-021-90248-w)
Supplement: Supplementary file 4 — Supplementary Information 1. [file 41598_2021_90248_MOESM4_ESM.pdf]

Supplementary Materials for

Controllable Multichannel Acousto-Optic Modulator and Frequency Synthesizer Enabled by Nonlinear MEMS Resonator

This file contains

Supplementary Notes 1-3
Supplementary Figures 1-5
Supplementary References 1-7
Supplementary Information of Videos 1-3

Supplementary Note 1

Finite Element Method and Device Modeling

Resonator Design

In a MEMS system that is supplied with a continuous driving force or signal, there occurs a steady conversion of energy from one form to another. The rate of energy transfer is frequency dependent and it reaches its local maxima at a certain operational point called the resonant frequency of the device and each resonant frequency corresponds to a unique vibration pattern known as the mode shape. COMSOL Finite Element Method (FEM) software was utilized to run Eigen-frequency analysis to identify the modes of interest and carry out frequency domain studies to extract the dynamic parameters. The flexural mode discussed in this work is the flapping mode[1] as shown in Fig. S1(a). Analytically[1] the resonant frequency of device A is 670kHz.

The material property of PZT used in the COMSOL simulation are quoted below.

(1) Density: 7750[kg/m^3]
(2) Elasticity matrix in stress form: {1.20346e+011[Pa], 7.51791e+010[Pa], 1.20346e+011[Pa], 7.50901e+010[Pa], 7.50901e+010[Pa], 1.10867e+011[Pa], 0[Pa], 0[Pa], 0[Pa], 2.10526e+010[Pa], 0[Pa], 0[Pa], 0[Pa], 0[Pa], 2.10526e+010[Pa], 0[Pa], 0[Pa], 0[Pa], 0[Pa], 0[Pa], 2.25734e+010[Pa]}
(3) Relative Permittivity matrix: {919.1, 919.1, 826.6}
(4) Coupling matrix: {0[C/m^2], 0[C/m^2], -5.35116[C/m^2], 0[C/m^2], 0[C/m^2], -5.35116[C/m^2], 0[C/m^2], 0[C/m^2], 15.7835[C/m^2], 0[C/m^2], 12.2947[C/m^2], 0[C/m^2], 12.2947[C/m^2], 0[C/m^2], 0[C/m^2], 0[C/m^2], 0[C/m^2], 0[C/m^2]}

At the target resonant mode, the resonator has a three segmented displacement profile along the width (W) of the device and its edges and central section are always out of phase. The z-displacement, phase and also strain across the AA′ cross-section plane is shown in Fig. S1(a). As the pseudo nodal line due to the mode shape passes along the side electrode regions, the amplitude of vibration would be lesser along segment I and segment III compared to segment II (central electrode). Polytec LDV measurement of the out-of-plane vibration of the side and central electrode area corroborates the simulated trend of z-axis displacement of the resonator. The resonant frequency is majorly a function of structural dimension along with the material property. The resonator used for the HHG study is a rectangular shaped TPoS structure with anchors at the central point of the width dimension. The major structural volume is composed of Single Crystal Silicon oriented along the <100> axis. The resonant frequency deviation trend with changes in the resonator's lateral dimension and also the material thickness is shown in Fig. S1(b) and (c) respectively. Generally, as the percentage volume of high acoustic velocity material increases, the resonant frequency moves to a higher value[2]. Nonlinear behavior of the resonator system is also thickness dependent as increasing the silicon thickness improves the power handling of the device[3] since the maximum energy (E_{max}) stored by the device is a function of stiffness as shown in the equation.

$$E_{\max} = \frac{1}{2}kx^2 \qquad (1)$$

where k and x are resonator's stiffness and vibration amplitude respectively. For a linear system, the stiffness is a unity powered; however, for a nonlinear system, the stiffness expression comprises higher order terms[4].

Butterworth Van Dyke Model of the resonator

A mass-spring-damper lumped model is a traditional method to represent a vibratory mechanical system. A resonator is a mass and spring system and to account for the energy losses, a damper is included as shown in Fig. S2(a). For an input force *F*, the resultant displacement *x* and its time derivatives can be represented as

$$m_{eff}\frac{\partial^2 x}{\partial t^2}+b_{eff}\frac{\partial x}{\partial t}+k_{eff}x=F \tag{2}$$

where m_{eff}, b_{eff} and k_{eff} represent the effective mass, damping, and stiffness of the system. An electrical equivalency can be developed from the mass-spring-damper system[5]. Hence, the output response for an applied voltage *V(t)* can be expressed as

$$L\frac{di(t)}{dt}+i(t)R+\frac{1}{C}\int i(t)dt=V(t) \tag{3}$$

where *L*, *R*, *C*, and *i(t)* are the electrical inductance, resistance, capacitance, and the generated current respectively.

A lumped element electrical circuit that characterizes a MEMS resonator is commonly known as the Butterworth Van-Dyke (BVD) model[6]. The BVD model has two branches namely motional and static arms as shown in Fig. S2(b). Apart from the constituent materials, the motional arm components are generally mode dependent, unlike the static arm element which is majorly a function of the area of the active electrode. The feedthrough capacitance (C_f) is caused by the interaction between the input and output electrodes. The capacitances (C_{O1} and C_{O2}) are the individual port capacitance between the top and bottom electrodes. The two-arm BVD model transfer function fits for a single resonant frequency and the feedthrough level around it. At the resonant frequency, effective impedance falls to a minimum value of motional resistance (R_m), as the motional capacitance (C_m) and inductance (L_m) impedances cancel out each other. The resonant frequency is hence computed by equating both motional capacitance impedance (X_{Cm}) and motional inductance impedance (X_{Lm}). The equations used for the calculation of the BVD model elements are also listed below.

$$R_m=2*Z_0(10^{\frac{I.L.}{20}}-1) \tag{4}$$

$$L_m=\frac{QR_m}{\omega_r} \tag{5}$$

$$C_m=\frac{1}{\omega_r^2 L_m} \tag{6}$$

where Z_0, *I.L.*, *Q,* and ω_r are the termination impedance, insertion loss at resonance, quality factor, and the resonant frequency in radian respectively. Fig. S2(c) shows the fitted frequency response of the BVD model and the resonator measurement in the air for a driving power of -25 dBm. The parameters are tuned around the values attained using the above equations to fit the measurement data.

<u>Supplementary Note 2</u>

Nonlinear Piezoelectricity Theory

For a resonator operating in the low amplitude drive non-hysteresis region, the device follows the linear piezoelectricity principle. Following the first law of thermodynamics, conservation of energy for the linear piezoelectric continuum can be represented as[7]

$$\dot{U}=T_{ij}\dot{S}_{ij}+E_i\dot{D}_i \tag{7}$$

where *U* is the stored energy density for the piezoelectric continuum and *T*, *S*, *E* and *D* are the stress, strain, electric field, and electric displacement vector component respectively. The general constitutive equations for piezoelectricity in the linear regime can be expressed as

$$\begin{bmatrix} T \\ D \end{bmatrix} = \begin{bmatrix} c^E & -e^t \\ e & \varepsilon^S \end{bmatrix} \begin{bmatrix} S \\ E \end{bmatrix} \tag{8}$$

where *c, e,* and *ε* are elastic, piezoelectric, and dielectric constants, respectively and superscript *t* indicates transpose of the matrix. The abovementioned equations presented in the IEEE Standard of Piezoelectricity are valid only for low amplitude drive conditions. However, when the device moves into the nonlinear regime the piezoelectric constitutive equations have to be modified. N. Aurelle *et al.* provides equations that extend into the nonlinear domain[8]. Two nonlinear coefficients, ζ, and η are introduced to accommodate the extent of variation of Duffing and output amplitude respectively. Hence a non-matrix form of equation (8) can be written as

$$T = cS + eE + \zeta S^2 + \eta SE \tag{9}$$

As the output displacement spectra exhibit harmonics, the displacement *x(t)* can be expressed as

$$x(t) = \sum_{n=-\infty}^{n=+\infty} B_n e^{jn\beta t} \tag{10}$$

where β is driving pulsation. Using equations (9) and (10) in the mass damper equation of a transducer, the following equation can be deduced[7]

$$M\frac{\partial^2 x(t)}{\partial t^2} + 2\lambda\omega\frac{\partial x(t)}{\partial t} = -TA \tag{11}$$

$$\begin{aligned} &-\beta^2 \sum_{n=-\infty}^{n=+\infty} n^2 B_n e^{jn\beta t} + \omega^2 \sum_{n=-\infty}^{n=+\infty} B_n e^{jn\beta t} + \frac{\zeta A}{l^2 M} \sum_{n=-\infty}^{n=+\infty} \sum_{m=-\infty}^{m=+\infty} B_n B_m e^{j(m+n)\beta t} + j2\lambda\omega\beta \sum_{n=-\infty}^{n=+\infty} nB_n e^{jn\beta t} + \\ &\frac{\eta A E_o}{2lM}\left(\sum_{n=-\infty}^{n=+\infty} B_n e^{j(n+1)\beta t} + \sum_{n=-\infty}^{n=+\infty} B_n e^{j(n-1)\beta t}\right) = \frac{-eAE_o}{2M}\left(e^{j\beta t} + e^{-j\beta t}\right) \end{aligned} \tag{12}$$

where *M*, *A,* and *l* are the mass, area and length respectively of the transducer, ω is the pulsation-related to the linear system's natural resonant frequency and λ is the damping factor.

Supplementary Note 3

Nonlinear Measurements

Frequency Response

An alternating electric field is applied to the input electrode of the device over a range of frequencies. The indirect piezoelectric effect converts the input drive signal to mechanical displacement. At resonant frequencies of various flexural or bulk modes, the effective displacement is enhanced by a direct proportionality relation to the quality factor of the mode. The displacement-induced strain results in charge generation due to the direct piezoelectric effect, which is then collected by the output electrode. A wide frequency sweep of Design A for different drive amplitude is presented in Fig. S3(a) using the measurement setup shown in Fig. 7(a) of the main text. The extent of nonlinearity is stronger for flexural modes than the higher frequency bulk mode. The out-of-plane device deformation of flexural and bulk modes measured using the LDV for linear operation drive amplitude is shown in Fig. S3(b). The mode of interest exhibits a hardening phenomenon i.e., maximum amplitude overhangs to the higher frequency side with an increase in driving amplitude.

HHG Measurement

Fig. 4(b) in the main text, presents the HHG result in the electrical domain for Design B when driven at $2V_{pp.}$ To corroborate the result, mechanical displacement readouts were performed for the same driving configuration for a range of input signal amplitude including $2V_{pp}$ AC input as shown in Fig. S4. As the

drive amplitude increases, enhancement in the HHG phenomenon is seen as anticipated. The highest frequency peak corresponding to the 77th harmonics can be observed at 24.81MHz. As the maximum measurable frequency in the LDV facility is 25MHz, harmonics beyond 25MHz cannot be recorded. For the high amplitude drive of 2V_{pp}, the fundamental mode displacement gets into the over-range of the LDV sensor head. Hence to capture the mode shapes of the fundamental, second and third harmonic modes of the resonator, the device is driven at 200mV_{pp} and the mode shapes thereby captured is shown in Fig. 3(c) in the main text. Movies S1, S2, and S3 are the animation files of the out-of-plane displacement of Device B.

As a confirmatory test for the fact that the highly nonlinear resonant mode of the released MEMS PZT TPoS resonator is the source of the HHG phenomenon, two schemes were followed. Firstly, a sample of Design A is manually broken such that only the Ground-Signal-Ground pads of the two-port measurement scheme are left. A drive signal with an amplitude of 2V_{pp} was provided to the probing pads. As expected, there were no harmonics generated as can be seen in Fig. S5(a). The low output power shows that no signal is transmitted between the input and the output ports in the absence of the resonator and that the nonlinear phenomenon observed earlier solely comes from the released TPoS MEMS resonator. Next, to validate that the existence of a highly nonlinear resonant mode is quintessential for the HHG, Design A is driven at a far frequency offset from its flapping mode resonance. The output spectrum corresponding to the faded red region in Fig. S5(b) shows that no higher harmonics were generated as the output amplitude was very low, which shows it is difficult to drive the device into nonlinearity. Whilst when driven along the resonance region (faded blue region), a strong HHG phenomenon can be observed. The above two sets of measurements emphasize the fact that HHG comes from released TPoS MEMS and that it is necessary to drive the resonator at or in the close vicinity of the mode which has a high degree of nonlinearity.

Supplementary Figure 1

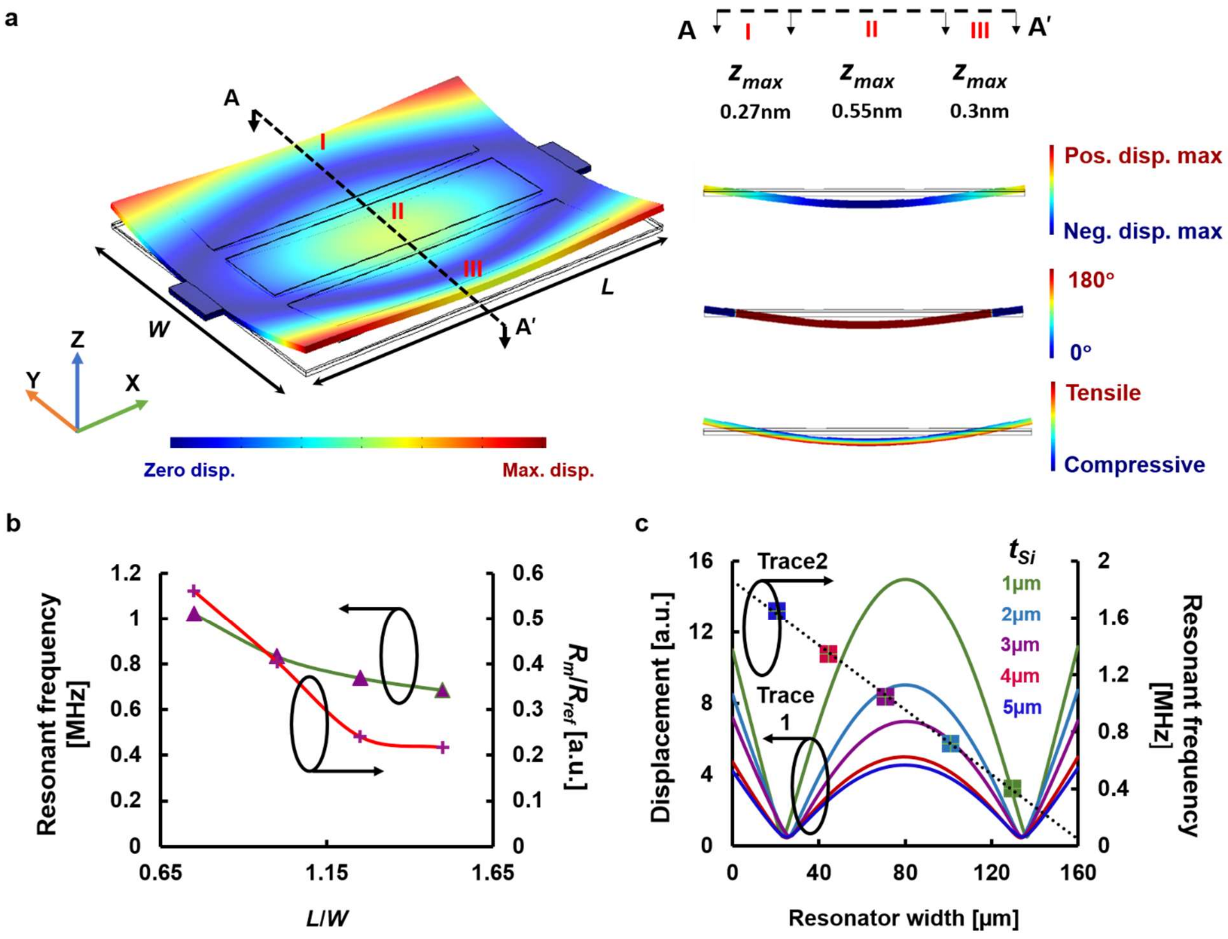

Fig. S1. Finite Element Modeling: a. *A three-dimensional plot of the total displacement of a rectangular resonator operating at its fundamental flapping mode. Total displacement is the summation of individual displacements along each X, Y, and Z-axis. Out-of-plane displacement's normalized magnitude and phase and also the strain along the width dimension for the AA' cross-section of the resonator.* ***b.*** *Increasing the resonator geometry (L- Length and W-width) leads to larger transduction and hence it reduces the motional resistance of the resonator and also enlarging the resonator geometry lowers the resonant frequency.* ***c.*** *Enhancing the thickness of silicon (t_{Si}) increases the overall Young's modulus and the effective stiffness of the resonator. Trace 1 shows the change in the deflection across the resonator width for different thicknesses of Silicon. Trace 2 shows the change in resonant frequency for varying thickness of Silicon.*

Supplementary Figure 2

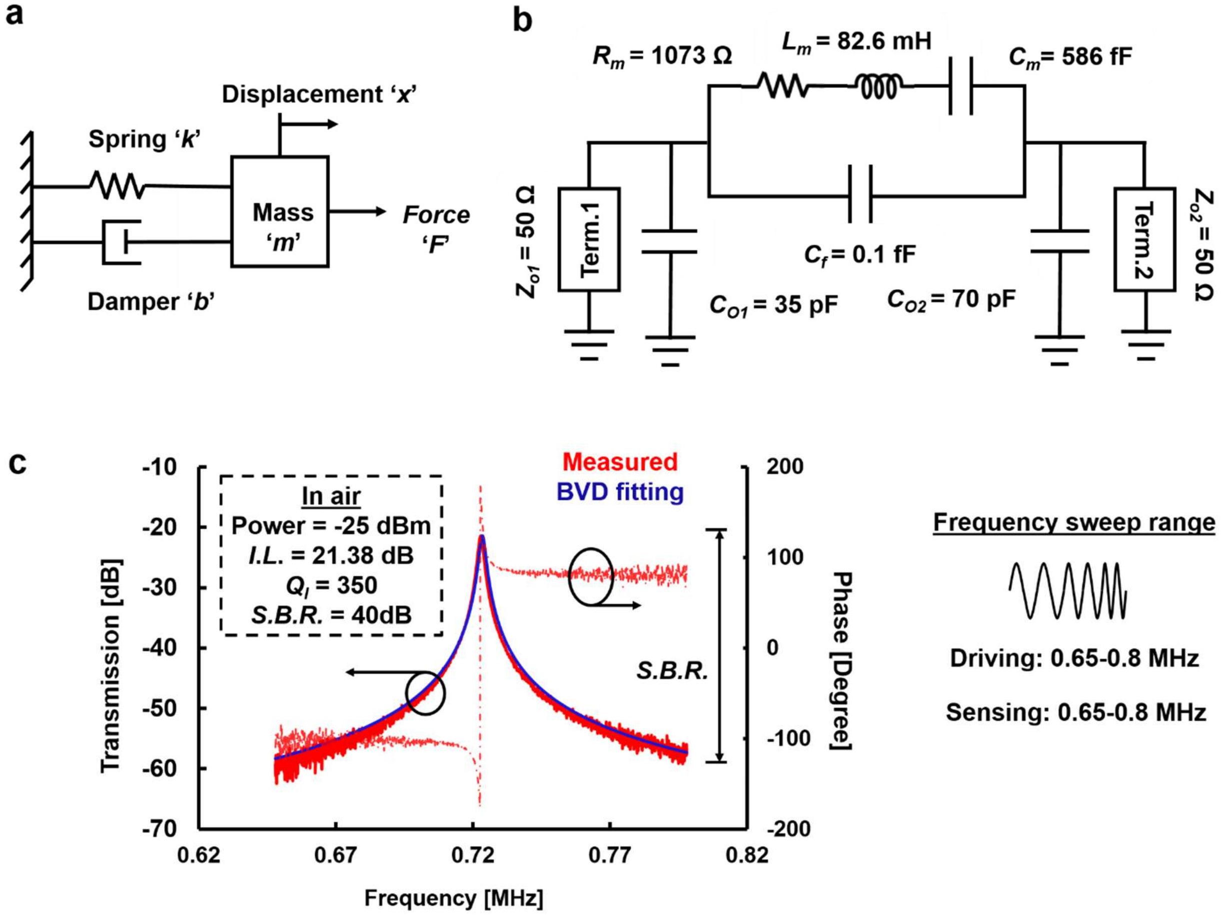

Fig. S2. Lumped resonator model: a. *Mass-spring-damper schematic of a mechanical vibratory system. The lumped arrangement of m, b, and k represents the resonator's mass, system losses, and the restoring force respectively.* ***b.*** *An electrical equivalency of the TPoS microelectromechanical resonator comprises Motional arm:* R_m, L_m, *and* C_m *and Static arm:* C_f. *The termination impedance in this work is 50Ω.* ***c.*** *The measured and fitted two-port transmission plot of the resonator operating in air for a driving power of -25 dBm. The phase information of the resonator measured using the Network Analyzer shows an overall 180º phase change at resonance.*

Supplementary Figure 3

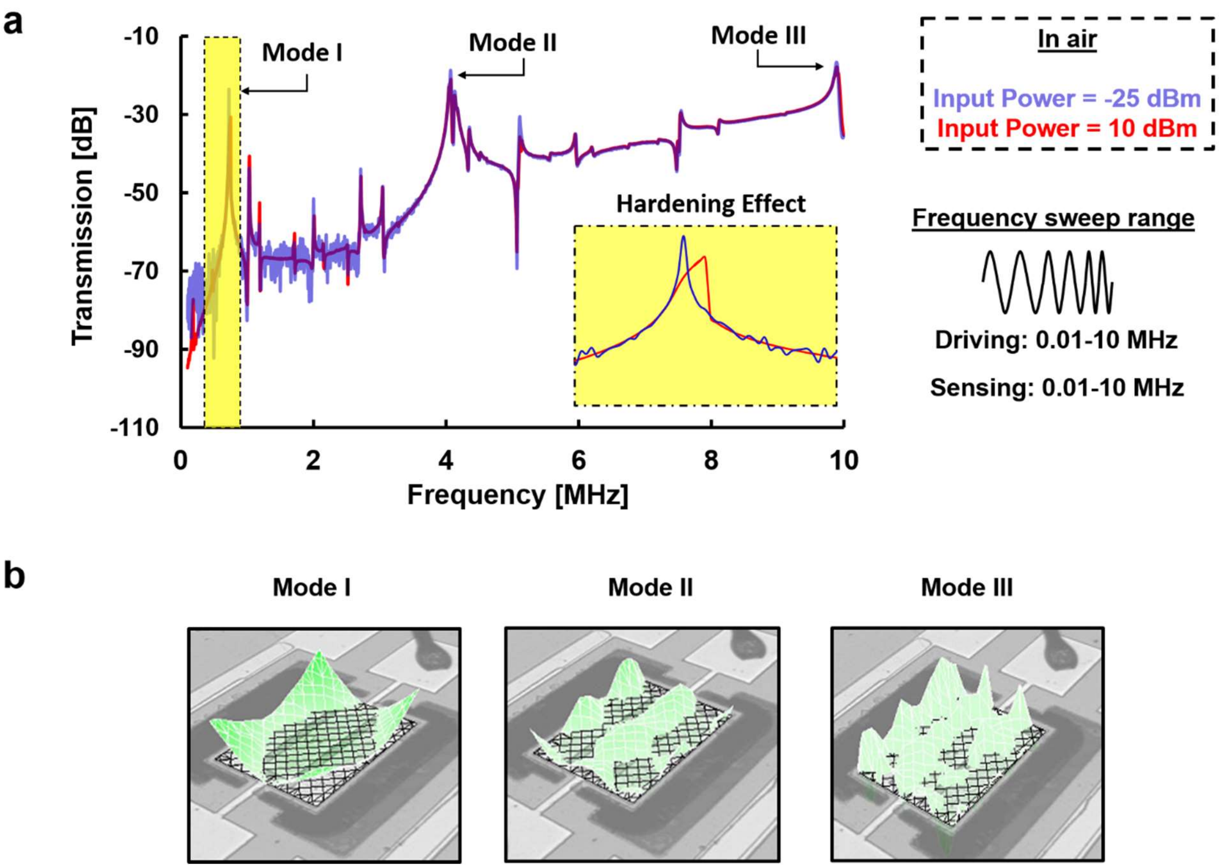

***Fig. S3. Wide sweep frequency response for low and high drive signal: a**. The drive and sense frequency range of the resonator is identical in this case i.e., from 100kHz to 10MHz. Many modes are generated due to the high electromechanical coupling property of the PZT thin film. The area highlighted in light yellow color is the frequency response of the mode of interest. A zoomed view of the transmission plot for varying drive levels is shown as an inset. StrongDuffing phenomenon can be observed for high driving power. **b**. The out-of-plane mode shapes of the significant peaks are acquired using Polytec LDV measurement system while the resonator is operated in the linear regime.*

Supplementary Figure 4

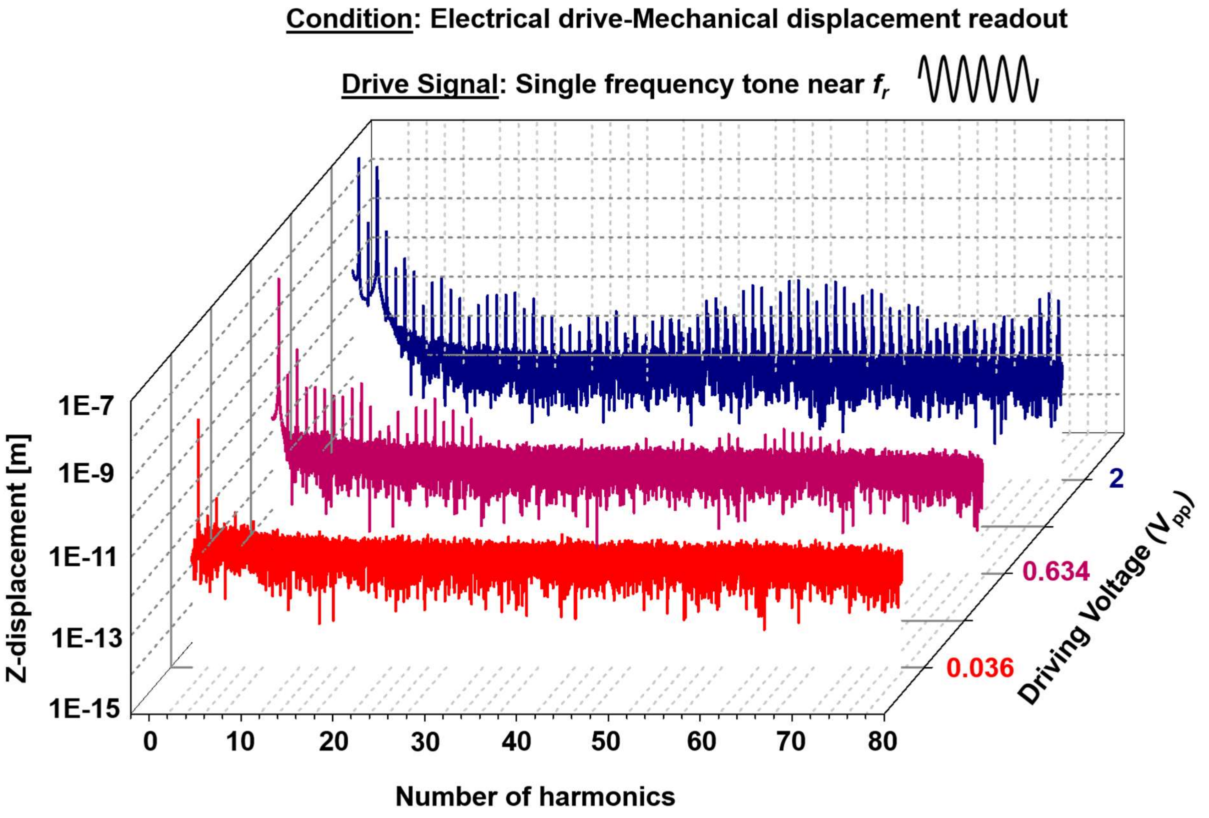

Fig. S4. HHG mechanical displacement readout: *Higher harmonic generation observation in Device B for input voltages of 0.036, 0.634, and 2*V_{pp}*. For this sample of the resonator, the fundamental flapping mode occurs at* f_r*=322kHz. The function generator output (i.e., the input signal to the resonator's central electrode) is a single tone frequency close to* f_r *and the output frequency of the LDV is swept from 100kHz to 25MHz.*

Supplementary Figure 5

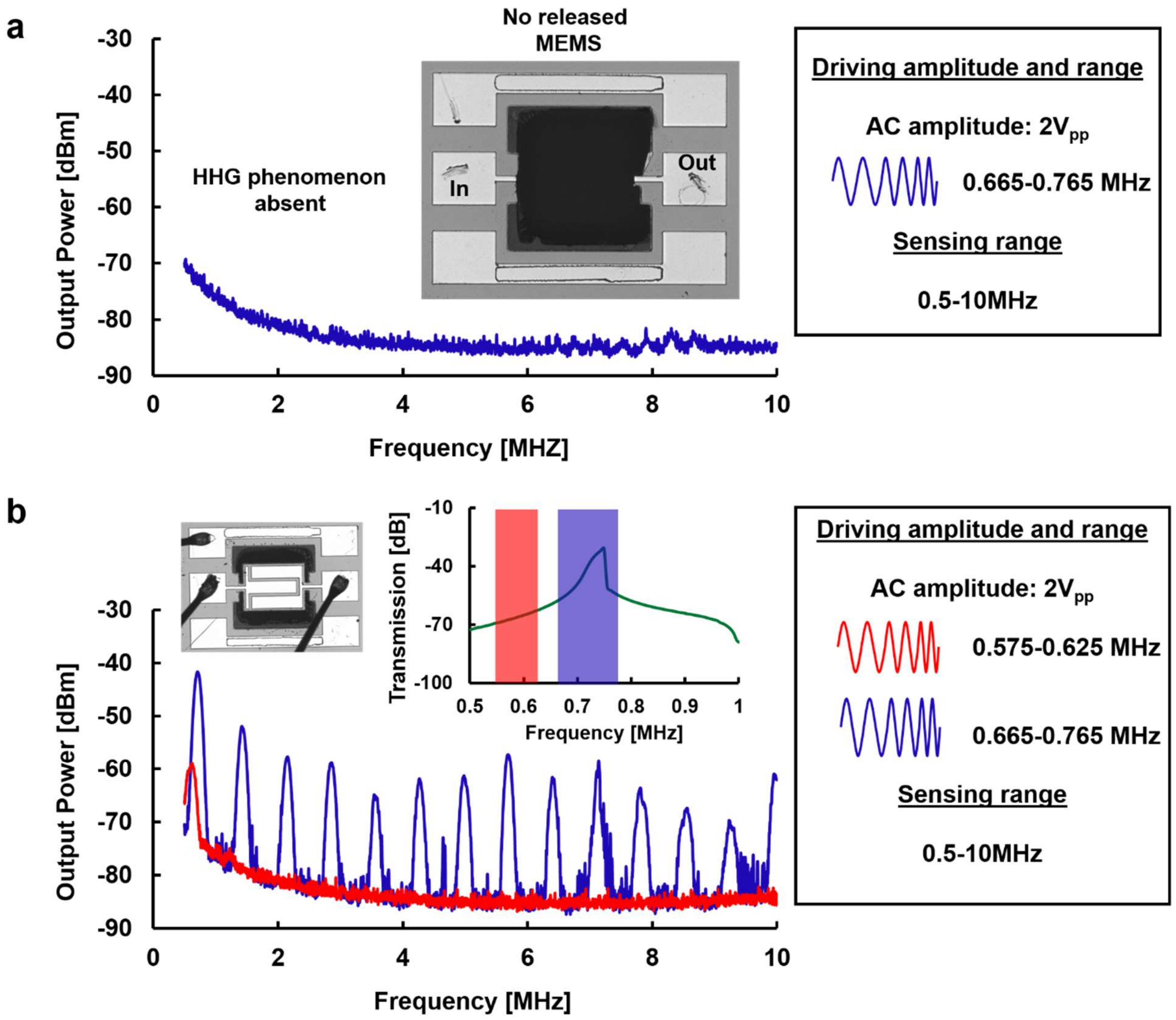

Fig. S5. Confirmation of HHG source: *The resonator is configured in a low-stiffness drive configuration for an AC input signal of 2*V_{pp} *and is subjected to different ranges of frequency sweep signal.* ***a****. The drive frequency is swept around the flapping mode resonant frequency and the output frequency span is set from 0.5-10MHz. The absence of peaks in the transmission spectrum of the Spectrum Analyzer with no released TPoS MEMS resonator between the driving and sensing ports indicates that there is no HHG phenomenon for this scenario. The inset shows the optical microscope image of the device under test.* ***b.*** *Spectrum Analyzer output power spectrum shows HHG when the resonator is driven along the nonlinear regime of the flapping mode, and no HHG when driven significantly far from resonance. The inset shows the Device A under measurement and different drive regions highlighted in the frequency response of the resonator measured using the Network Analyzer.*

References

1. Ghosh, S. & Lee, J. E.-Y. Piezoelectric-on-Silicon MEMS Lorentz Force Lateral Field Magnetometers. *IEEE Transactions on Ultrasonics, Ferroelectrics, and Frequency Control* **66**, 965-974 (2019).

2. Chandrahalim, H., Bhave, S.A., Polcawich, R.G., Pulskamp, J.S. and Kaul, R. PZT Transduction of High-Overtone Contour- Mode Resonators. *IEEE Trans. on Ultrasonics, Ferroelectrics, and Frequency Control* **57**, 2035-2041 (2010).

3. Abdolvand, R., Lavasani, H.M., Ho, G.K. and Ayazi, F. Thin-film piezoelectric-on-silicon resonators for high-frequency reference oscillator applications. *IEEE Trans. on Ultrasonics, Ferroelectrics, and Frequency Control* **55**, 2596-2606 (2008).

4. Elshurafa, A.M., Khirallah, K., Tawfik, H.H., Emira, A., Aziz, A.K.A. and Sedky, S.M. Nonlinear dynamics of spring softening and hardening in folded-mems comb drive resonators. *Journal of Microelectromechanical Systems* **20**, 943-958 (2011).

5. Dufour, I., Heinrich, S.M."Fundamental Theory of Resonant MEMS Devices" in *Resonant MEMS: Fundamentals, Implementation, and Application*, O. Brand, I. Dufour, S. M. Heinrich, F. Josse, Eds. (Wiley-VCH, 2015), ed. 1, chap. 1.

6. Horsley, D., Lu, D. Y., Rozen, O."Flexural Piezoelectric Resonator" in *Piezoelectric MEMS Resonators*, H. Bhugra, G. Piazza, Eds. (Springer, 2017), ed. 1, chap. 6.

7. An American National Standards, "IEEE Standard on Piezoelectricity," in ANSI/IEEE Std 176-1987 (https://ieeexplore.ieee.org/stamp/stamp.jsp?tp=&arnumber=26560).

8. Aurelle, N., Guyomar, D., Richard, C., Gonnard, P. and Eyraud, L. Nonlinear behavior of an ultrasonic transducer. *Ultrasonics* **34**, 187-191 (1996).

Supplementary Movie files

Movie S1.
Mode shape animation of the *z*-displacement recorded using LDV at the primary resonance mode i.e., 322.26kHz when the resonator is driven at f_d=322.26kHz using an AC signal of amplitude 200mV$_{pp}$. A relatively low value of drive signal is used for capturing the HHG mode shapes, such that vibration doesn't exceed the optimal displacement range of the LDV readout setup.

Movie S2.
Mode shape animation of the *z*-displacement recorded using LDV of the second harmonic resonance mode i.e., 644.43kHz when the resonator is driven at f_d= 322.26kHz using an AC signal of amplitude 200mV$_{pp}$.

Movie S3.
Mode shape animation of the *z*-displacement recorded using LDV of the third harmonic mode i.e., 966.69kHz when the resonator is driven at f_d=322.26kHz using an AC signal of amplitude 200mV$_{pp}$.
